# Structural Ceramics Modified by Water Treatment Plant Sludge

**DOI:** 10.3390/ma13225293

**Published:** 2020-11-23

**Authors:** Alexander Orlov, Marina Belkanova, Nikolay Vatin

**Affiliations:** 1 Institute of Architecture and Construction, Russian Federation, South Ural State University, 454080 Chelyabinsk, Russia; orlovaa@susu.ru (A.O.); belkanovami@susu.ru (M.B.); 2 Higher School of Industrial, Civil and Road Construction, Russian Federation, Peter the Great St. Petersburg Polytechnic University, 195251 Petersburg, Russia

**Keywords:** ceramic bricks, ceramic sintering, air shrinkage, WTP sludge, water treatment plants

## Abstract

Water treatment plant (WTP) sludge is actively used in building materials production. The object of this research was modifying additives for ceramic bricks from WTP aluminium-containing sludge. The research aim of this study was to determine the suitability of a million-plus population city’s WTP sludge as a burning-out additive in the production of structural ceramics and to establish the optimal conditions for obtaining products with the best characteristics. The raw water belongs to water belongs to the hydrocarbonate class, the calcium group, and it is of low turbidity (1.5–40 mg/L kaolin). Sludge, sourced from WTP sedimentation tanks, was dewatered by adding lime or by using the freezing-thawing method. The spray-dried WTP sludge is introduced into the clay in amounts of 5% to 20% by weight. The addition of 20% reduces the sensitivity of the clay to drying, reduces the density of ceramic by 20% and simultaneously increases its compressive strength from 7.0 to 10.2 MPa. The use of WTP sludge as a modifying additive, pretreated by the freezing-thawing method, makes it possible to obtain ceramic bricks with improved properties. The results can be used for WTP sludge containing aluminium obtained by treating water of medium turbidity and medium colour.

## 1. Introduction

Water treatment plant (WTP) sludge is actively used in the production of building materials, which reduces the consumption of natural resources, the environmental load and the cost of building materials [1,2,3,4]. The use of WTP sludge in building materials could exclude expensive and energy-intensive stages of sludge utilization. WTP sludge can be used in structural ceramic production [5,6,7,8,9,10,11,12]. The volume of sludge to the volume of WTP treated water varies from 0.1% to 1%, and in some cases, can reach 5% [13]. WTP sludge is formed when surface or ground water is clarified in sedimentation and clarification tanks. Such sludge is a gelatinous mass, with a humidity of 95% to 99%, and contains mineral and organic substances, products of coagulant hydrolysis, phytoplankton cells, and other components [14].

### 1.1. The Use of Water Treatment Plant Sludge in Building Materials Science

WTP sludge can be used in the production of building materials and products, such as ceramic bricks and tiles, expanded clay, Portland cement clinker, and lightweight concrete aggregate [2,3,6,7,15,16,17]. For these purposes, the sludge is dewatered and dried to a constant weight aggregate [2,3,6,7,15,16,17]. The article [16] proposes introducing WTP sludge to the composition of Portland cement in the amount of 4% to 7%. A further increase in the proportion of sludge sharply reduces the strength of the samples. The author points out that, in the samples, there is no leaching of heavy metals that were initially present in the raw sludge, confirming the environmental friendliness of this method of sludge disposal. According to [16], spray-dried sludge can be used in the amount of 12% in cement production, partially replacing clay and limestone. A decrease in the compressive strength does not allow the use of WTP sludge in the manufacture of structural concrete. More research is needed to assess the potential negative impact of sulfate ions on the long-term effectiveness of cement materials made with WTP sludge [17]. Sulfate ions lead to ettringite formation in concrete and, consequently, to a weakening of the structural integrity and changes in mechanical properties over time [17].

There are some advantages in the use of WTP sludge for the production of structural ceramics. First, it has a composition similar to clay in terms of inorganic oxides (oxides of aluminium, silicon, iron). Secondly, potentially toxic elements are immobilized during heat treatment into forms that are not prone to leaching [17,18]. Since WTP sludge contains a significant amount of finely dispersed organic substances [19], it can be used as a burning-out additive for structural ceramics. Burning-out additives increase the porosity and thermal resistance of ceramic bricks, the latter being one of their most important characteristics. Studies [20,21] show that WTP sludge can be used as an additive to reduce firing temperatures and as a red pigment. In [22], it was shown that the addition of WTP sludge enhances clay sintering and leads to the formation of mullite at lower temperatures.

Recommendations for dosage of sludge in different publications differ significantly from each other. On the one hand, the authors of [18] note a general decline of ceramic materials’ characteristics with the addition of WTP sludge. Flexural strength, water absorption, linear firing shrinkage, and apparent specific mass impair. Despite this, the authors recommend the addition of 10% WTP sludge for the production of solid bricks that meet Brazilian national standards. In [21,23], it is also proposed to add no more than 10% WTP sludge due to a decrease in the mechanical characteristics of products. On the other hand, in [23] it is noted that the addition of 40% WTP sludge and 5% processed tea waste allows to obtain fired clay bricks with improved thermal insulation properties and improved compressive strength.

The use of sludge as a modifying additive requires its preliminary dewatering. WTP sludge is a complex multicomponent system with a high content of coagulant hydrolysis products and a highly developed surface. The humidity as a result of sludge pumping exceeds 95% [18,24]. Such sludge, taking into account its hydroxide nature, cannot be filtered without preliminary treatment. The thawing method is recommended to increase its water-carrying capacity, reagent treatment (lime, flocculant and filler material) or freeze-thaw conditioning. After processing, the sludge is sent to filter presses for mechanical dewatering.

The properties of WTP sludge must be consistent for using them as a raw material in the construction industry. The quality of the water source is subject to seasonal fluctuations. Therefore, the dose of coagulant and other reagents varies. This variation leads to a change in the composition and the water-yielding capacity of WTP sludge. It is necessary to select an appropriate method to treat the sludge before dewatering.

Dewatered WTP sludge can be used for the production of structural ceramics. It can reduce the firing temperature and the density of the ceramics, i.e., work as a flux and burning-out additive. However, there are no standard recommendations for the use of WTP sludge in the production of structural ceramics in the literature.

### 1.2. Sludge Composition

Sludge obtained by the treatment of surface waters is classified according to the quality of the water source [13]. To do this, the ratio of the water colour index to water turbidity (WCI/WT) is determined. The higher this ratio, the more difficult it is to dewater the sludge, and additional treatment is required before its dewatering. At a WCI/WT ratio of more than 30, the sludge is hardly dewatered.

Sludge contains free and bound water. The latter differs in its degree of boundedness: first, water included in the composition of floccules; second, water bound to the particle surface by adsorbing and adhesive forces; and, finally, chemically bound (hydrated) water [25,26].

The technology of water treatment determines the composition and properties of WTP sludge, and the reagents used [14,17,27]. In most cases, water is treated with coagulants, that is, hydrolyzable salts of aluminium and iron. Therefore, it is common practice to distinguish sludge containing aluminium and sludge containing iron [14,27,28,29]. Coagulant hydrolysis leads to a high aluminium or iron hydroxide content. Due to its hydroxide nature, sludge has a low water-carrying capacity. The mineral component of WTP sludge is close to the composition of clay [15,16,30,31,32,33] and includes compounds of silicon, aluminium, iron, calcium and magnesium, sodium, and potassium [15]. In some cases, manganese and titanium are present in the amount of less than 1%. There are also indications that sludge contains zinc, cobalt, lead, cadmium, and nickel [34].

### 1.3. Aims and Objectives

There is no data in the publications on the influence of the preliminary preparation methods of WTP sludge for dehydration on the properties of building ceramics.

Because of that, the study aims to evaluate the effect of the WTP sludge preparation method on the properties of building ceramics. The research objectives are:

1. to choose the optimal method for preliminary sludge dewatering;
2. to establish the best method of sludge treatment on the properties of structural ceramics;
3. to determine the optimal amount of sludge introduced into the clay;
4. to determine the most effective firing temperature and establish the properties of the resulting ceramic bricks.

## 2. Materials and Methods

### 2.1. WTP Sludge Treatment

Aluminium-containing sludge from the WTP of Chelyabinsk, Russian Federation, was used in experiments as a typical WTP sludge of a city with a million-plus population. The source of city water supply is the Shershnevskoe Reservoir on the Miass River. In terms of its chemical composition, the reservoir water belongs to the hydrocarbonate class, the calcium group, and it is of low turbidity (1.5–40 mg/L kaolin). Depending on the season, it can be of medium and high colour (18–120 on Pt-Co scale). The average WCI/WT ratio of the source water during the period of sludge formation is 3.3, which makes it possible to classify the sludge by the quality of the source water as being of medium turbidity and colour.

The choice of the optimal conditions for the sludge treatment was made according to the samples obtained during the emptying of two-tier sedimentation tanks. If water treatment at the WTP uses aluminium sulfate coagulants, aluminium oxychloride coagulants, and AN-905 flocculant based on polyacrylamide, then the sludge classified as aluminium-containing.

Two methods were used to increase the water-yielding capacity of sludge. They are lime treatment and freezing-thawing. Freezing was carried out at −16 ± 2 °C. Samples of sludge with a volume of 1–1.5 L were frozen for seven days. Thawing was carried out at 20 ± 3 °C.

The lime treatment was carried out using powdered lime (PL) dosing and hydrated lime (HL) dosing. PL dosing reduces the filtrate volume during sludge dewatering. Dry lime with 43% active ingredient (CaO) was used. The lime was introduced in an amount of 15% to 40% of the dry sludge matter and was mixed for 30 min.

An aqueous suspension of lime was prepared to treat water with HL slurry. The content of the active part by CaO was determined. The hydrated lime was added at the rate of 10% to 15% of dry sludge matter and was stirred for 10 min.

**Figure 1:**
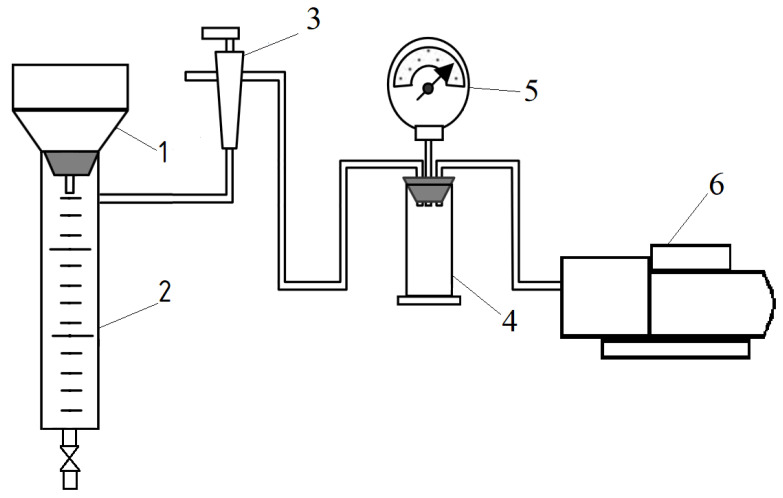
Diagram of a laboratory installation for dewatering and determining the specific filtration resistance. 1—Buchner funnel; 2—measuring cylinder; 3—shut-off valve; 4—receiver; 5—vacuum gauge; 6—vacuum pump.

After treating the sludge with lime or by the freezing-thawing method, water was removed from the sludge by vacuum pumping (67 ± 5 kPa) in a laboratory installation (Figure 1). The cake was dried to a constant weight at 105 ± 2 °C in a drying oven.

### 2.2. Experimental Testing

According to the Hartley method, two-factor experiment was carried out. Among varying factors were the amount of additive as a percentage of the clay weight (D%), and the firing temperature (T_O_, °C). The response indicators included plasticity number, shard density (ρ), air shrinkage, and fire shrinkage. To obtain reliable results, it turned out that two repetitions of each experiment were sufficient, while the experimental error was less than 5%. The properties of the clay and ceramic shards were determined following the requirements of the regulatory documents listed in Table 1.

The strength-density ratio (SDR) was calculated as the ratio of the sample density (ρ, g/cm^3^) to its compressive strength (R, MPa):

SDR = ρ/R.(1)

Preparation of specimens, air shrinkage and fire shrinkage tests were carried out in accordance with the Russian State Standard GOST 21216-2014 [35]. Specimens with dimensions of 60 × 30 × 10 mm were cut from the clay mass layer using a mould with a pusher. The specimens were marked at 50 mm intervals. The specimens were dried at a temperature of 100 °C for 24 h and were fired in a laboratory electric oven.

The heating rate during the drying process was kept constant at 3 °C/min. The samples were kept at the final temperature for 30 min, in accordance with the requirements of the Russian State Standard GOST 21216-2014 [35].

To determine the ultimate compressive strength, cube specimens with dimensions of 50 × 50 × 50 mm were prepared and dried at a temperature of (105 ± 5) °C to constant weight. The samples were fired in a laboratory electric oven.

The heating rate during the drying process was kept constant at 3 °C/min. The samples were kept at the final temperature for 30 min, and the density was tested and determined by using a hydraulic press.

The sensitivity to drying was determined in accordance with Russian State Standard GOST 21216-2014 [35] by the duration of exposure to a heat flux on a freshly formed sample until cracks appeared on it.

The clay for the fabrication of samples was prepared at Kemma Ceramic Ware Plant, Chelyabinsk, Russia. 

## 3. Results

### 3.1. Sludge Treatment Methods

The traditional technology for the production of structural ceramics involves the introduction of dry modifying additives. However, WTP sludge has a high content of bound water. The value of the specific resistance to filtration (r, m/kg) characterizes the sludge’s water-carrying capacity. Studies [18,24] have shown that the untreated sludge of Chelyabinsk WTPs has a specific resistance to filtration between 1.5 × 10^13^ m/kg and 6.3 × 10^13^.

In the preliminary experiments, the use of powdered lime for reducing the specific resistance to filtration requires up to 30% and 40% of lime and a mixing time of 20 to 30 min (see Table 2). The resulting spray-dried sludge will contain up to 40% lime, which can have a significant effect on the properties of ceramic products and requires additional research.

The use of HL makes it possible to distribute the reagent through the sludge more evenly and to reduce the processing time to 10 min. Acceptable values of the specific filtration resistance are achieved with the introduction of 20% lime.

FT showed the best results for improving the water-carrying capacity of sludge and can be recommended as the best method for reducing the specific resistance to filtration of WTP sludge. After thawing, the initial value of 3.74 × 10^13^ m/kg decreased to 0.05 × 10^13^ m/kg. The moisture content of the sludge cake after thawing and vacuum pumping was 66.7%, while the cake without treatment had a moisture content of 96.2%.

### 3.2. Influence of the Sludge Treatment Method on the Properties of Ceramics

A comparative experiment was carried out to identify the influence of the sludge dewatering method on the properties of the resulting ceramics. For clay mixtures with the addition of FT treated WTP sludge (WTPS-FT) and HL treated WTP sludge (WTPS-HL), the plasticity number was determined, and for ceramic samples, the density of the shard, and the air and the fire shrinkage were determined. The sludge was introduced in the amount of 5% of the weight of the initial clay. According to published data [21,22,23], WTP sludge was introduced into clay in an amount of 5% to 40%. In this study, the minimum value for a preliminary assessment of the effectiveness of the WTP sludge was chosen. The scatter of results for plasticity number does not exceed 0.1%; air shrinkage does not exceed 0.05%; fire shrinkage does not exceed 0.01%; the density of ceramic shard does not exceed 0.01 g/cm^3^. Each test was repeated twice to obtain reliable results. Firing was carried out at 950 °C. The results are shown in Table 3.

Table 3 shows both additives significantly reduce the air shrinkage of the sample from 9.7% to 8.0%. This positive effect arises due to the presence of a significant amount of natural organic matter in the sludge [34]. The introduction of additives will reduce the tendency of the raw material to crack and accelerate drying.

The fire shrinkage of the samples with WTPS-FT additive is 15% higher than the samples without additives. This increase in shrinkage indicates an increase in the degree of sintering, which will increase the strength and frost resistance of the samples. This effect is associated with the presence of fluxing agents (FeO, K_2_O and MnO) in the WTPS-FT additive [37]. The presence of fluxing agents in the dry matter of the sludge is 3% in total, which corresponds to 0.15% of the total weight. About 40% of the mass is lime in the WTPS-HL additive, so the fluxing agent content decreases. The addition of WTPS-HL does not improve ceramic sintering.

An increase in shrinkage indicates an increase in sintering, but the bulk density decreases. This effect can be explained by the fact that WTPS-FT has a dual effect. On the one hand, WTPS-FT is a burnout additive. Burnout of organic matter in the sediment creates additional porosity, which leads to a decrease in density. On the other hand, the WTPS-FT additive has a fluxing effect due to the oxides (FeO, K_2_O and MnO) present. With an increase in the amount of WTPS-FT additive in the clay batch, the density of the ceramic decreases more than the shrinkage increases.

### 3.3. Selection of Optimal Conditions for Sludge Pretreatment

Both additives reduce the density of the ceramic shard due to the burnout of the organic component; however, the WTPS-HL additive contains a significant amount of hydration in the Ca (OH)_2_ lime composition. Water vapour, evaporating during lime dehydration, contributes to the porosity of the shard and a significant decrease in its density to 1.48 g/cm^3^ in comparison with the samples with the WTPS-FT additive.

The WTPS-FT additive has a complex positive effect, improving the properties of the clay, raw material and ceramic sample. WTPS-FT has the properties of a plasticizer, a blowing agent, and a flux. Therefore, further studies were carried out with FT pretreated sludge.

### 3.4. The Influence of the Additive Dosage and Firing Temperature on the Structural Properties of the Ceramics

A two-factor experiment was carried out using the Hartley method to determine the effect of the WTPS-FT additive on the properties of the clay and ceramic shard. The design matrix and the results are presented in Table 4. The amount of WTPS-FT additive introduced varied from 5% to 10% of the weight in increments of 2.5%. Each test was repeated twice to obtain reliable results. The scatter of results for air shrinkage does not exceed 0.05%, for fire shrinkage does not exceed 0.01%, and for the density of ceramic shard does not exceed 0.01 g/cm^3^.

**Figure 2:**
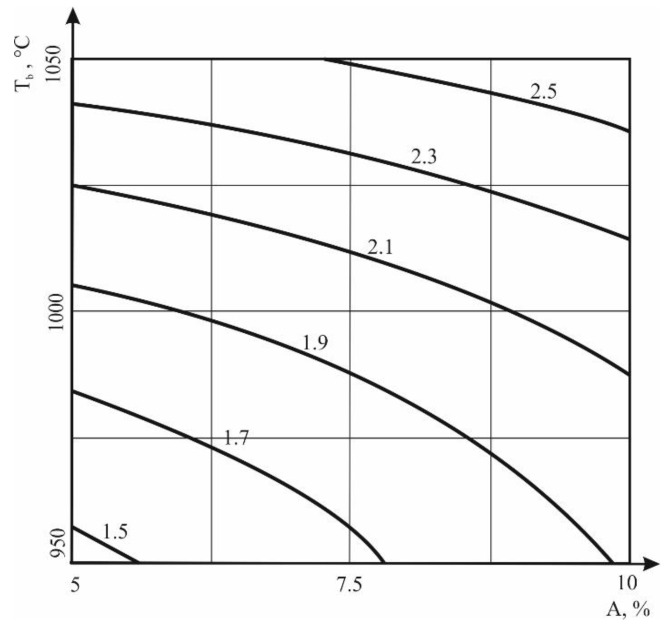
Dependence of fire shrinkage (FS) on the amount of additive in the clay and the temperature of raw firing. Equation of the dependency graph: FS = 2 + 0.17x + 0.43y + 0.01x^2^ − 0.06xy + 0.09y^2^.

**Figure 3:**
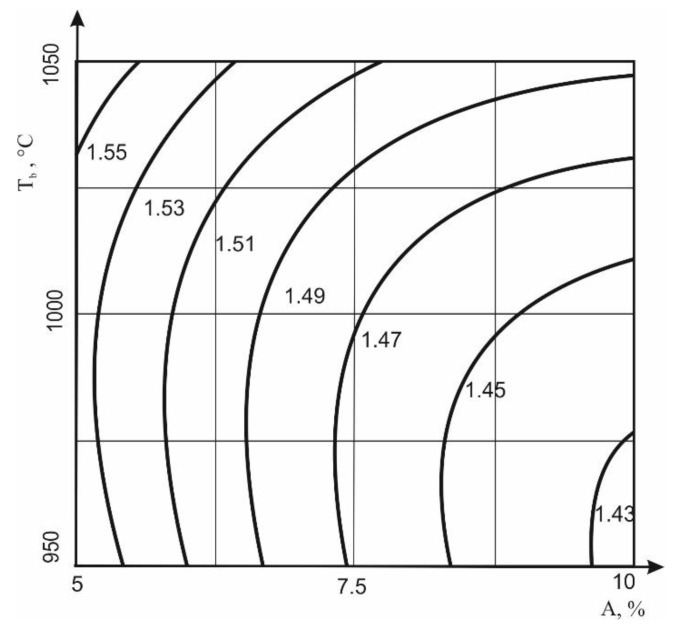
Dependence of the shard density on the amount of additive in the clay and the firing temperature of raw material. Equation of the dependency graph: ρ = 1.47 − 0.05x + 0.02y + 0.02x^2^ + 0.01xy + 0.02y^2^.

An increase in the additive dose expands the range of permissible operating humidity, since the plasticity number increases from 15.5% to 16.9%. In addition, an increase in the percentage of additive from 5% to 7.5% leads to a decrease in air shrinkage from 8% to 7.6%. The isolines for the fire shrinkage and the density of the ceramic shard are presented in Figure 2 and Figure 3. The equations describing the graphs of dependencies are placed under the corresponding figures.

The Fisher coefficient is 2.7, which is lower than the table value (4.3). This confirms the adequacy of the model.

The nature of the dependence in Figure 2 indicates that an increase in the amount of additive leads to an increase in fire shrinkage, that a larger amount of melt is formed, and that the ceramic is sintered more efficiently. The effect of the additive is especially significant at lower firing temperatures. The introduction of the additive allows a reduction in the firing temperature by 50 °C without deteriorating the degree of sintering.

The Fisher coefficient was 2.5, which is lower than the table value (4.3). Low value of the Fisher coefficient confirms the adequacy of the model.

The addition of WTPS-FT reduces the density of the ceramic shard due to the burnout of organic compounds in the sludge (Figure 3). The porous effect of the additive is most pronounced at lower firing temperatures, since an increase in temperature contributes to a larger amount of melt.

The combination of the porous and fluxing action of the additive can significantly change the quality of the ceramics. With a decrease in the density of ceramics, at the same time, an increase in fire shrinkage is observed. The study hypothesized that due to the increase in fire shrinkage, the strength of the sample would increase. Further experiments confirmed this hypothesis. In order to confirm this assumption, an additional experiment was carried out in the laboratories of Kemma Ceramic Ware Plant, Chelyabinsk, Russia. WTPS-FT was used as a modifying additive. The samples were fired at a temperature of 1000 °C, since this temperature corresponds to the technology of the enterprise. The following properties of the samples were controlled: air and fire shrinkage, sensitivity to drying, density and the compressive strength of the ceramic shard. Each test was repeated three times to obtain reliable results. The scatter of results for sensitivity to drying does not exceed 10 s; for air shrinkage does not exceed 0.05%; for fire shrinkage does not exceed 0.05%, and for density of ceramic shard does not exceed 0.03 g/cm^3^. The results of the experiment are presented in Table 5. Dosages were agreed with Kemma Ceramic Ware Plant as a potential consumer of the additive being developed. The upper limit (20% of the additive of the clay weight) is determined by the economic feasibility and technical properties of the resulting ceramics.

The data presented in Table 5 confirm previous experiments (see Table 3 and Table 4). The introduction of WTPS-FT into the clay improves the properties of the ceramic samples. The amount of the additive increases up to 20%, which leads to an increase of the ceramics’ strength and a decrease in the density. On the one hand, the WTPS-FT additive has a fluxing effect and increases the strength of the ceramic. This regularity is confirmed by an increase in fire shrinkage with an increase in the amount of additive. On the other hand, WTP-FT porosizes ceramics, and therefore porosity increases. However, the fluxing properties of WTPS-FT lead to the formation of denser pore walls, sintering of the bulk of the ceramic body.

The high sensitivity of the clay to drying is a significant technological problem. Such clays require longer drying and are prone to cracking. Table 5 shows that the introduction of WTPS-FT into the clay reduces the sensitivity of the raw material to drying, which means the clay is characterized as insensitive (Russian State Standard GOST 21216-2014 [35]). The use of the WTPS-FT additive reduces the drying time and the defect rate during production. These changes may be associated with the presence of polyacrylamide in WTPS-FT, which is part of the flocculant used in the water treatment.

## 4. Conclusions

The article studies the applicability of WTP sludge in the production of structural ceramics. A method for the preliminary treatment of sludge, making it possible to effectively dewater the sludge, and use it to obtain high-quality structural ceramics, is proposed. The sludge was prepared using powdered lime (WTPS-PL), hydrated lime (WTPS-HL) or the freezing-thawing method (WTPS-FT). The effects of adding WTP sludge on the characteristics of ceramic products and the optimal amount of additive are established. To select the most effective treatment method, the authors evaluated its effect on the properties of the resulting ceramics. The most effective method is freezing-thawing. It should be noted that the freezing-thawing method of sludge treatment before dewatering is implemented on some WTPs [28].

A two-factor experiment varied the amount of sludge in the clay and the firing temperature. The introduction of the WTPS-FT additive made it possible to reduce the density, and the firing temperature of ceramics by 50 °C, without loss of sintering. The testing was carried out at the laboratory of Kemma Ceramic Ware Plant, Chelyabinsk, Russia.

The utilization of WTP sludge in the production of structural ceramics is possible. The introduction of sludge helps to reduce the density of structural ceramics and increase its strength-density ratio. A significant advantage of the WTPS-FT additive is a decrease in the sensitivity of the clay to drying, leading to a reduced drying time and defect rate.

The results obtained are compatible with the following conclusions:

1. The utilization of WTP sludge in the production of ceramic bricks is promising.
2. A 20% addition of WTP sludge reduces the sensitivity of the clay to drying, reduces the density of ceramics by 20% and increases its compressive strength from 7.0 to 10.2 MPa.
3. The use of WTP sludge as a modifying additive, pretreated by the freezing-thawing method, makes it possible to obtain ceramic bricks with improved properties.
4. The results can be used for WTP sludge containing aluminum, obtained by treating water of medium turbidity and medium colour.
5. The results of the study confirmed the possibility of using WTP sludge on an industrial scale for the production of high-quality ceramic bricks.

## Figures and Tables

**Table 1:** Testing methods.

Property	Regulatory Document
Plasticity number of clay mass	Russian State Standard GOST 21216-2014 [35] Clay raw materials. Test methods, p. 5.3
Air shrinkage of clay sample	Russian State Standard GOST 21216-2014 [35] Clay raw materials. Test methods, p. 5.26
Fire shrinkage	Russian State Standard GOST 21216-2014 [35] Clay raw materials. Test methods, p. 5.27.4.3
Compressive strength of ceramic sample	Russian State Standard GOST 21216-2014 [35] Clay raw materials. Test methods, p. 5.33.4
Sensitivity of clay sample to drying	Russian State Standard GOST 21216-2014 [35] Clay raw materials. Test methods, p, п. 5.32
Density of ceramic shard	Russian State Standard GOST 7025-1991 [36] Ceramic and calcium silicate bricks and stones. Methods for water absorption and density determination and frost resistance control, p. 5

**Table 2:** Resistivity of sludges treated with powdered lime (PL) and hydrated lime (HL).

Treatment Method	Specific Resistance to Filtration, × 10^13^ m/kg					
	Lime%					Sludge without Treatment
	10	15	20	30	40	
PL	–	–	2.34	1.16	0.77	8.40
HL	1.28	1.10	0.880	–	–	3.74

**Table 3:** Comparison of clay properties.

Property	Without Additives	With WTPS-FT Additive	With WTPS-HL Additive
Mixing moisture content%	34.8	30.0	32.8
Plasticity number%	15.0	15.5	13.9
Air shrinkage%	9.7	8.0	8.0
Fire shrinkage%	1.4	1.6	1.4
Density of ceramic shard, g/cm^3^	1.67	1.53	1.48

**Table 4:** Design matrix and responses of the two-factor experiment.

First Factor, X		Second Factor, Y		Plasticity Number%	Shrinkage		Density of Ceramic Shard, g/cm^3^
Code Values	D%	Code Values	T_O_, °C		Air Shrinkage%	Fire Shrinkage%	
−1	5.0	−1	950	15.5	8.0	1.60	1.53
0	7.5	−1	950	16.8	7.6	1.70	1.47
1	10.0	−1	950	16.9	7.5	1.90	1.42
−1	5.0	0	1000	-	-	1.86	1.53
0	7.5	0	1000	-	-	2.02	1.47
1	10.0	0	1000	-	-	2.15	1.45
−1	5.0	1	1050	-	-	2.43	1.57
0	7.5	1	1050	-	-	2.46	1.51
1	10	1	1050	-	-	2.69	1.49

**Table 5:** Strength of ceramic samples.

Sample	Sensitivity to Drying, Seconds	Air Shrinkage%	Fire Shrinkage%	Density of Ceramic Shard, g/cm^3^	Compressive Strength Limit, MPa	Strength-Density Ratio
Without additives	100	6.2	1.5	1.57	7.0	4.2
With 10 % of WTPS-FT additive	>180	4.5	2.5	1.45	8.5	5.6
With 20 % of WTPS-FT additive	>180	4.2	3.5	1.33	10.2	7.7
